# In Vitro Assays: Friends or Foes of Cell-Penetrating Peptides

**DOI:** 10.3390/ijms21134719

**Published:** 2020-07-02

**Authors:** Jinsha Liu, Sepideh Afshar

**Affiliations:** Protein Engineering, Lilly Biotechnology Center, Eli Lilly and Company, San Diego, CA 92121, USA; liu_jinsha@lilly.com

**Keywords:** cell-penetrating peptides (CPPs), in vitro assays, penetration, internalization, translocation, plasma membrane, lipid vesicle membrane

## Abstract

The cell membrane is a complex and highly regulated system that is composed of lipid bilayer and proteins. One of the main functions of the cell membrane is the regulation of cell entry. Cell-penetrating peptides (CPPs) are defined as peptides that can cross the plasma membrane and deliver their cargo inside the cell. The uptake of a peptide is determined by its sequence and biophysicochemical properties. At the same time, the uptake mechanism and efficiency are shown to be dependent on local peptide concentration, cell membrane lipid composition, characteristics of the cargo, and experimental methodology, suggesting that a highly efficient CPP in one system might not be as productive in another. To better understand the dependence of CPPs on the experimental system, we present a review of the in vitro assays that have been employed in the literature to evaluate CPPs and CPP-cargos. Our comprehensive review suggests that utilization of orthogonal assays will be more effective for deciphering the true ability of CPPs to translocate through the membrane and enter the cell cytoplasm.

## 1. Introduction

The cell membrane is an asymmetric phospholipid bilayer with aminophospholipids mostly at the inner (cytoplasmic) leaflet and cholinephospholipids preferentially at outer (exoplasmic) side [1,2]. Proteins that are embedded in the lipid bilayer can facilitate signal transduction or function as transporters, enzymes, or joining proteins. The key function of the cell membrane is to create a physiological barrier allowing compartmentalization and to tightly regulate cell entry. As a result, exogenous compounds including therapeutic peptides, antibodies, siRNA, and nanoparticles cannot easily access the inside of the cell that harbors 60–70% of the human proteome [3]. To overcome this challenge, cell-penetrating peptides (CPPs) are exploited as versatile delivery vehicles to cross cell membrane.

CPPs are cationic, amphipathic, or hydrophobic peptides of 5–39 amino acid in length [4,5]. The most widely studied CPPs are truncated version (Tat_48–60_ and Tat_49–57_) of trans-activator of transcription (Tat) protein from HIV-1 [6,7,8,9] and penetratin (16 residues) from the homeodomain of *Drosophila antennapedia* [10,11,12]. Energy-independent direct translocation and energy-dependent endocytosis are generally accepted as the main internalization mechanisms of CPPs [13,14,15]. Nevertheless, the mechanisms of CPP-cargo cell entry have remained controversial. One point of view is that the same CPP can employ multiple cell entry mechanisms depending on the peptide local concentration, avidity, cell membrane lipid composition, and cell type [16,17,18]. However, alterations in physiochemical properties of a peptide, even as slight as conjugation to a dye, has been shown to influence the mechanisms and efficiency of the uptake [19]. This has led to the second point of view that CPP and cargo are one unit where the physiochemical properties of the CPP, the cargo (fluorescence dye or any functional moiety), and their combination can influence delivery pathways and their final destination. 

Among over ten thousand CPPs described in the literature, only a few Tat-based therapeutic peptides have reached the advanced stages of clinical trials [8,9,20]. Utilization of CPPs as “trojan horses” has been limited due to their entrapment in the endosomes upon cell entry [21,22,23]. This limitation promoted a tremendous effort to optimize the potency of CPP-cargos and the discovery of new CPPs with greater innate and specific delivery performance inside the cell [23,24,25,26]. However, difficulties in discriminating cytoplasmic uptake from endosomally trapped molecules have hampered the identification of true CPPs for therapeutic purposes. These difficulties have also limited our understanding of the physiochemical parameters that determine final intercellular localization of CPP-cargos. Therefore, extensive attempts have been made to develop cell- and lipid vesicle-based assays that can determine cell entry and cytoplasmic localization. These assays that include intracellular fluorescence detection by microscopy and/or flow cytometry, transcriptional reporter system, and mass spectrometry are discussed in this review [4]. Critical factors in assay development, such as material generation and sensitivity of detection should be also taken into careful consideration. In this review, in vitro cell-based assays and an in-depth review of recent strategies to utilize lipid vesicles for study of CPPs are discussed. Advantages and limitations of each approach are also summarized. Table 1 presents an overview of CPPs mentioned in this review paper. 

## 2. Cell-Based In Vitro Assays

Cellular internalization of the CPP can be detected using three strategies where (1) CPP is conjugated to a tag or a functional moiety, (2) mammalian cells are engineered to express a specific protein that is used to detect cytoplasmically localized CPP, or (3) the combination of first and second approaches is utilized. In this section, intracellular detection of fluorescence by microscopy and flow cytometry, protein complementation, phenotypic alteration induced by a functional group, and transcriptional reporter system are discussed. Gene delivery approaches are also examined to highlight the recent advancement in assay development. The main focus of all these assays is to distinguish CPPs that enter cytoplasmic domain of the cell from the ones that are entrapped in the endosomes.

### 2.1. CPPs as Vehicles of Biotin or Fluorescent Entities

Laser scanning confocal fluorescence microscopy and flow cytometry have been heavily used to quantify cellular uptake of fluorochrome or biotin conjugated CPPs [50,51,52,53]. Fluorescent tags and biotin can be conjugated by the standard N-hydroxysuccinimide (NHS), isothiocyanate, or click chemistry to peptides [54,55,56]. One limitation of direct conjugation such as NHS is the inability to control distribution and quantity of dyes on a single peptide. Thus, the high intensity of fluorescence inside the cell may be misleading and might be due to low concentration of CPP with high amount of conjugated dye. To improve this, site-specific labeling methods were developed. For example, phage coat protein such as PIII or PVIII, was engineered to display a sequence containing LPE***T***G, a recognition motif for prokaryotic enzyme Sortase. Sortase cleaves after a Thr residue and forms an intermediate with the phage, which is resolved upon covalent bond formation with α-amine of a fluorophore labeled poly-glycine peptide. As the result, a site-specific fluorophore labeling is achieved [57]. Such site-specific labeling techniques can be employed in CPP studies to improve intracellular uptake quantification. 

Assays that use dye-labeled peptides with quenching models were revised to reduce non-specific interaction. For example, highly fluorescent 7-nitrobenz-2-oxo-1,3-diazol (NBD) fluorophore was used to label CPPs such as penetratin. NBD is irreversibly inactivated in to nonfluorescent 7-amino-2,1,3-benzoxadiazol-4-yl in the presence of dithionite [58]. Internalization of NBD-penetratin was tested in human leukemia K562 cells at either 37 °C or 4 °C, followed by a treatment of dithionite at 4 °C. Any portion of the CPP that was not internalized (extracellular and membrane bound) was inactivated upon addition of the cell impermeable dithionite at 4 °C, hence fluorescence was only due to the internalized peptide [27]. In a different study, Hallbrink and colleagues labeled the CPP with a fluorescence quencher 3-nitrotyrosine (CPP-nitroY-C) and coupled it to a non-penetrating cargo (LKANL) bearing the 2-amino benzoic acid (Abz) fluorophore (Abz-C-LKANL) via a disulfide bond. Penetratin, transportan, Tat, or MAP (KLAL) were used as CPPs. The cellular uptake of Abz-C-LKANL was indicated as an increase in fluorescence intensity when the disulfide bond in the construct (CPP-quencher-S-S-cargo fluorophore) was reduced in the intracellular domain due to the reducing environment [28]. The internalization assay was performed in 96-well plates with application of peptide complex at concentrations ranging 0.1–10 µM in Bowes human melanoma cells. Dithiothreitol (DTT) was added to the wells to reduce external peptide-cargo and the overall fluorescence intensity was used as the max value. The internalized CPP-S-S-cargo was measured proportionally as fractions to the total amount of constructs added. Fluorophore-quencher pair such as TAMRA-QSY7 [59] has also been employed to monitor polypeptides entry into endosomal vesicles where activated fluorescence, caused by dequenching, was measured. Considering the low pH and reducing environment of the endosomal and lysosomal vesicles and the fact that fluorescence intensity was measured by a spectrometer in these studies, final destination of CPP-cargo remains undefined and additional work is needed to verify endosomal escape of CPPs.

The use of fluorescent tags, although the primary method to detect peptide and peptide-cargo inside the cells, has many limitations. Direct dye-attachment may create artifacts due to degradation and self-quenching in low pH environment such as endocytic vesicles [17]. The fluorescent dyes and biotin, conjugated to CPPs, do not share similar properties as therapeutic cargos. Therefore, effectiveness of CPPs as vehicles of therapeutic cargos remains somewhat unclear [60]. Detection of the internalized CPPs by confocal imaging has its own limitations. First, certain methods of cell fixation might cause artificial signal or re-localization/redistribution of the fluorescent dye [61], if immunostaining is conducted on fixed cells. Second, the microscopic detection is strongly dependent on the accessibility of antibody to the target and/or streptavidin to biotin inside the cell. Measurements of fluorescent intensity might be influenced by the use of automatic or manual intensity scaling, causing a misleading signal of uptake. The drawbacks of cell fixation and antibody accessibility can be overcome by using live cell imaging to detect and monitor the uptake of the fluorescence tagged CPP. In order to differentiate cytosolic entry of CPP-cargo, fluorescent cell-permeable trackers are needed to outline the cytosolic domain from other organelles. This approach requires a careful evaluation to avoid nonspecific background signals, physiological artifacts, cytotoxicity, and most importantly, any interference between the cell-permeable trackers and CPP-cargo entry.

Continuous innovation in the field of protein chemistry can have a positive impact in the CPP-related research. Fluorescent proteins with photoswitching behavior in acidic compartments compared to the cytosol have been used to study autophagic flux and endocytosis in transfected mammalian cells. Ratiometric response induced by the pH sensitive fluorescent proteins can be measured in different cellular compartments by fluorescent microscopy and/or flow cytometry. For example, pH-stable enhanced GFP (eGFP) was fused to a pH-sensitive RFP [62,63] to detect protein levels in various cellular compartments. Similarly, fusion of a pH-stable cyan fluorescent protein (FP) variant mTurquoise2 to a highly pH-sensitive enhanced yellow fluorescent protein eYFP (pH-lemon) [64] had enabled protein quantification in different compartments. In acidic cellular compartments such as endosomes and lysosomes, yellow fluorescence would be reduced while the cyan fluorescence would be enhanced due to the reduced Forster resonance energy transfer (FRET) efficiency. Thus far, the pH sensitive probes have not been employed as cargos of CPPs and their function in the context of CPP is yet to be determined. 

### 2.2. CPPs as Vehicles of Split Protein for Fragment Complementation

Few functional proteins can be dissected into two inactive fragments. These conditional split-protein assembly was developed with ubiquitin, dihydrofolate reductase (DHFR), β-lactamase, TEV protease, chorismite, thymidine kinase, firefly luciferase, and green fluorescence protein (GFP) [65]. Fluorescence or luminescence complementation is the most common strategy to reconstitute a functional protein upon spatial proximity of the two split parts. 

**Figure 1:**
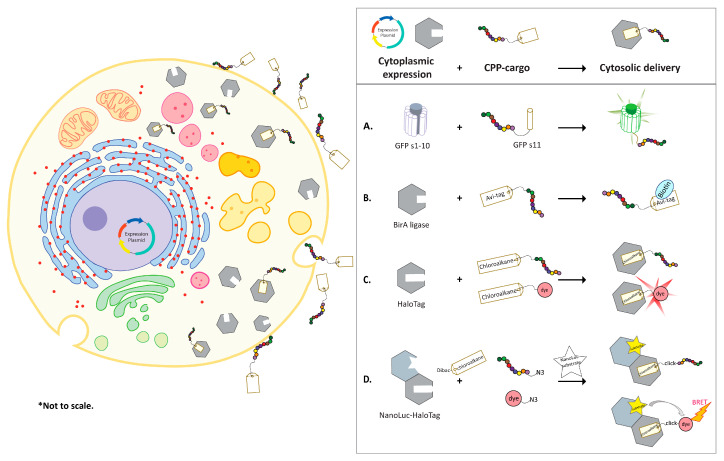
Schematic of CPPs as vehicles of split protein and functional tags. Mammalian cells are engineered to express a specific protein in cytoplasmic domain. The cargo delivery is facilitated by CPP and detected in cytoplasmic by (**A**) Split-GFP complementation assay, (**B**) BirA-based cytosolic delivery assay, (**C**) Chloroalkane penetration assay, and (**D**) NanoClick assay. Dibac: Dibenzoazacyclooctyne; N3: azide-modification (click, click-reaction by Dibac and azide functionalized molecules).

GFP and its variants such as eYFP have been extensively optimized to enhance solubility, reconstitution efficiency, and to reduce background signals [66,67]. In the natural state, the proteins are assembled into a barrel shaped structure composed of 11 strands of β-sheets that allows peptidyl backbone cyclization and formation of a fluorescence chromophore. GFP can be split between strand seventh and eighth [68] or strand tenth and eleventh [67,69,70]. Similarly, eYFP can be split between the seventh and the eighth [71,72], eighth and the ninth [73,74], or tenth and the eleventh β-sheet strands [75,76]. Co-incubation of the two fragments of GFP in trans results in efficient reconstitution of GFP chromophore bond and restores GFP fluorescence. Smaller complementary fragments, such as strand 11 (s11) of the GFP have been used as cargos of CPPs while the larger fragments were engineered for cytoplasmic expression in mammalian cells (Figure 1A). The split assembly ensures that complementation occurs only when the CPP-GFPs11 enters the cytoplasmic area to form the full length GFP molecule [77,78,79]. The split GFP assay offers several advantages; it confirms true cytoplasmic localization through GFP complementation fluorescence; cytoplasmic entry can be monitored in real-time and in a quantitative manner by flow cytometry, and it can easily be adapted to a high-throughput assay format. Sensitivity of the assay can be influenced by the expression level of GFPs1–10 fragment in the cytosol. As a result, signal threshold in individual cells caused by GFP fluorescence depends on the number of CPP-cargo delivered inside the cells and the expression level of GFPs1–10 fragment. 

### 2.3. CPPs as Vehicles of Functional Tags

Cytosolic localization of a CPP can be validated if its cargo undergoes an enzymatic reaction that occurs solely in the cytoplasm. In this approach, substrate conversion and/or product formation should be easily detected. Utilization of multi-functional tags (SNAP-, CLIP- and ACP-tags) [80,81,82] that have been used for labeling soluble or cell surface proteins has been limited in CPP evaluation due to their large size and bulky structure. On the other hand, few known short tags that act as a specific enzyme substrate have poor efficiency. For example, serine in the 11-residue peptide “ybbR tag” (DSLEFIA***S***KLA) was identified as a substrate for Sfp phosphopantetheinyl transferase [83] with only 17% labeling efficiency [84]. Characteristics of the tag, enzymatic efficacy, substrate specificity, and feasibility of labeling inside mammalian cells is consequential in this approach. This section will describe three technologies for evaluation of CPPs that have escaped endosomal entrapment. All technologies exploit an enzymatic reaction that occurs in the cytosol. 

Verdurmen and colleagues developed a biotin ligase-based assay to measure cytosolic delivery [85,86,87]. Biotin ligase (BirA) biotinylates lysine residue in the Avi-tag (GLNDIFEAQ***K***IEWHE) [88]. Various cells lines, such as SKBR3, MCF7, HT29, and HEK293 were engineered to stably overexpress *E. coli* derived BirA in the cytosol [89] (Figure 1B). Avi- and HA-tagged CPPs carrying eGFP (Avi-HA-CPP-eGFP) were added to the BirA expressing cells. Cells were then lysed, and the cytosolic delivery was quantified by western blots using fluorescently labeled streptavidin relative to the uptake of the HA tag. The design was improved by Hoffmann and co-workers with an additional step of adding sodium pyrophosphate (PPi) to stop the BirA reaction prior to cell lysis and streptavidin capture. The platform was applied in discovery of novel CPPs from a random peptide library displayed on T7 phage [90]. The cytoplasmic biotinylation differentiated CPPs from the peptides that did not internalize or were trapped in the endosomes. As a result of ten independent selection campaigns using different cell lines, thousands of CPPs were identified. These novel CPPs were evaluated in cell-based screening assays such as GFP complementation assay. Hits were optimized for potency and half-life extension [90]. 

The second technology utilizes HaloTag to interrogate cytosolic localization in chloroalkane penetration assay (CAPA) [29,91]. Halo-tag in the cytosol of cells can be covalently conjugated to any chloroalkane conjugated CPPs, if the CPP-cargo has found its way to the cytosol [92,93,94]. This is followed by the addition of a chloroalkane-dye for binding to the unreacted free HaloTag. The dye intensity reversely correlates to the cytosolic CPP levels and could be quantified by flow cytometry (Figure 1C). Chloroalkane tagged Tat, penetratin and nona-arginine (9R) showed concentration-dependent cytosolic localization after 4 hours with CP_50_ values at 3.1, 0.82, and 0.3 µM, respectively. CP_50_ was defined as the concentration at which 50% cell penetration was observed under assay conditions. The CP_50_ values were comparable to values obtained for the same CPPs in the assay where MALDI-MS was used to detect internalized peptides [50]. 

The third strategy, NanoClick, was developed by combining the HaloTag technology and in-cell copper-free Click chemistry. The cytosolic uptake of azide-tagged CPPs was monitored quantitively by a NanoBRET signal in cells [95]. In this assay, Dibenzoazacyclooctyne-chloroalkane (DiBac-CA) was applied to cells expressing NanoLuc-HaloTag in the cytoplasmic domain. This was followed by application of azide-modified CPPs to anchor the intracellular HaloTag. Subsequent introduction of azido-dye and NanoLuc substrate to cells allowed the detection of a BRET signal that reversely correlated with the concentration of the azide-CPP in the cytosol (Figure 1D). 

All strategies are great approaches to detect CPPs that are localized to cell cytoplasm. Special attention should be paid to ensure that internalization and cytoplasmic localization are due to the CPP and not the tag, as tags may also lead to potential artifact due to degradation. Different linkers, connecting CPP to its cargo, may also have an effect on the molecule penetration. Both assays, mentioned above, require cell line engineering to overexpress enzymatic proteins in the cytosol. Hence, the sensitivity of the assays might be limited by protein expression level. Moreover, transfection might be challenging for some cell lines of interest, such as primary cells. 

### 2.4. CPPs as Vehicles of Functional Groups to Enable Phenotypic Readouts

Various functional peptides or proteins that could attenuate protein-protein or enzyme-substrate interactions were fused to the classical CPPs, particularly Tat and penetratin, to evaluate their potential as vehicles for intracellular delivery [96]. The phenotypic changes induced by CPP-cargo internalization can be categorized as gene activation or blocking, cellular cytotoxicity or protection, or simply fluorescence/luminescence emission. Nearly three decades ago, Fawell and co-workers chemically cross-linked Tat peptides to cell-impermeable entities such as β-galactosidase, horseradish peroxidase (HRP), RNase A, and domain III of *Pseudomonas* exotoxin A (PE). The intracellular uptake of Tat-facilitated β-galactosidase and HRP were measured colorimetrically by histological staining, and the uptake of Tat-RNase A and -PE were evaluated by the induced cytotoxicity in HeLa cells [97]. In this section, we discuss a selected number of functional groups that can be used as a cargo to allow specific readouts in the cell-based assays. Smaller size cargos are discussed first. 

Apoptosis induced by the cargo is the most commonly used readout to evaluate CPPs. A synthetic 14-amino acid pro-apoptotic peptide KLAKLAKKLAKLAK (KLAKLAK)_2_ was shown to disrupt the mitochondria membrane at 10 µM and cause cytotoxicity. This cationic peptide was fused to peptide CNGRC or RGD-4C (ACDCRGDCFC) to facilitate tumor cell entry [98,99]. A similar approach was carried out by Chen and collaborators, where two octa-peptides, PVKRRLDL and PVKRRLFG derived from E2F1 cell-cycle regulatory transcription factor family, were fused to Tat or penetratin. Both peptides were shown to block phosphorylation of E2F by cyclin/cdk2. Internalization of the peptides, facilitated by fusion to Tat or penetratin, resulted in deregulation of E2F, inhibition of cdk2, and cellular cytotoxicity in U2OS osteosarcoma and MDA-MB-435 cancer cells [37].

Inhibition of intracellular phosphorylation, as the result of CPP-cargo cell entry has served as a frequent readout. The eleven residue STAT1 binding peptide derived from measles virus V protein (MV-V) with the sequence YHVYDHSGEAV (MV-V_110-120_) was reported by Caignard and co-workers. The peptide was fused to Tat and octa-lysine (8K) for intracellular delivery to inhibit STAT1 phosphorylation [38]. When localized to cytoplasm of target cells, both Tat-MV-V_110–120_ and 8K-MV-V_110–120_ were able to block type I interferons signaling pathway by approximately 40%. Peptide-based STAT3 inhibitors are also reported [100,101]. The inhibitory phospho-peptides (P***Y***LKTK and ***Y***LPQTV, phosphorylation on Y) bind to the SH2 domain of STAT3 and block dimerization, however, low potency (mM concentration) have limited their further use. Kim and colleagues identified a new STAT3 binding peptide with the sequence HGFQWPG(SWTWENGKWTWK)GAYQFLK. Fusion of this peptide to the N-terminus of 9R [44] resulted in reduced STAT3 phosphorylation in a DNA-binding assay and suppressed cell viability and proliferation in cancer cells. Alternatively, it was shown that cells could be rescued from apoptosis by CPP fusion to survival peptides. For example, C-Jun N-terminal kinase (JNK) binding peptide (RPKRPTTLNLFPQVPRSQDT) when fused to Tat, referred as D-JNK-1, protected βTC-3 cells from stress-induced apoptosis [39]. D-JNK-1 blocked the phosphorylation of c-Jun activation domain in the cytoplasm, prevented the formation of transcription complexes, and inhibited cell death in β- and hair-cells [39,40]. 

An enzymatic assay was developed to assess cytosolic entry of ubiquitin-PEP-Alexa Fluor 594 conjugated to the C-terminus of Tat. Cytoplasmic deubiquitinating enzymes (DUBs) act on Tat-ubiquitin-PEP-Alexa 594 to release PEP-Alexa 594, which can be detected by fluorescent microscopy and by the reduction in size in SDS-PAGE [41]. Limitations include low throughput assay format and the need for a secondary detection step such as western blot. 

Engineered bacteriophage has been used as cargos of CPPs, in which an Avi-tag was displayed on PIX coat protein of phage. Ypep peptide (YTFGLKTSFNVQ) was displayed on PIII protein to facilitate cell penetration [46]. In vitro biotinylation by BirA generated a biotin tagged phage on PIX protein allowing streptavidin conjugated HRP to bind to phage. The whole complex was introduced to PC-3 human prostate cancer cells for intracellular delivery and the uptake of Ypep-phage-HRP was detected colorimetrically by incubating cells with the substrate TMB (3,3′,5,5′-tetramethylbenzidine). No significant cell cytotoxicity was observed in the group of Ypep-phage-HRP (5 × 10^10^ pfu/mL). Subsequently, cells were treated with indole-3-acetic acid (IAA). IAA interacted with HRP to induce cell death only in cells that were first treated with Ypep-phage-HRP. In this approach, phage served as a nanocarriers for exogenous protein delivery with the advantages of easy manipulation and amplification. 

Development of functional moieties that allow phenotypic readouts due to CPP cell entry has been a challenging task. First, utilization of a functional group might alter biophysical and chemical properties of CPP-cargo or mask the function of the CPP. Depending on the functional group used, cellular assays need to be optimized and different cell types need to be engineered. Moreover, the efficacy of the assay is shown to be highly dependent on the function and sensitivity of the particular peptide/protein. Functional peptides normally require a µM–mM concentration for detectable alteration [102], making material generation a challenge.

### 2.5. CPPs as Vehicles of Translocation Cassettes Activators

Gene-based reporter assays have been developed to monitor both cell entry and nuclear translocation. Cre recombinase was fused to Tat peptide and introduced to *lox*P-STOP-*lox*P-eGFP expressing cells. In the event of cell penetration and nucleus translocation of Tat-Cre, the STOP DNA segment would be excised, leading to eGFP expression [23]. Yu and colleagues developed a luciferase based transcriptional reporter assay to monitor relative cell permeability of CPPs in 96-well plates. OxDex-activated ester, an agonist of the glucocorticoid receptor (GR) was conjugated to the N terminus of the CPP (OxDex-CPP). A cell line such as HeLa was co-transfected with two plasmids; one for encoding a fusion protein comprised of Gal4 DNA-binding and dimerization domains (DBD) and a VP16 transactivation domain (Gal4DBD-GRLBD-VP16), and the second plasmid for encoding a Gal4-driven firefly luciferase gene. In the event of OxDex-CPP conjugates entering the intracellular domain, OxDex would bind to GRLBD and release the Gal4DBD-GRLBD-VP16 complex from heat shock protein 90 (Hsp90). As a result, fusion protein translocates to nucleus, binds to Gal4 binding site in the firefly luciferase gene and activates it. Subsequently, the expressed luciferase can be quantified [103,104]. Holub and co-workers improved the assay sensitivity by optimizing the affinity of GR variants to the steroid and replacing luciferase by eGFP for detection. As a result, two assays, glucocorticoid-induced eGFP induction (GIGI) and glucocorticoid-induced eGFP translocation (GIGT) were developed. In GIGI assay, eGFP expression is the readout and the amplified assay signal is detected by flow cytometry. In GIGT assay, reporter expression is bypassed such that GR-eGFP fusion protein in the cytosol would translocate to the nucleus upon binding to CPP-GR agonist. This assay requires sophisticated imaging equipment [105].

### 2.6. CPPs as Vehicles for Gene Delivery and Expression

Gene delivery by CPPs has been an attractive alternative to viral gene delivery. In 2003, plasmid DNA encoding luciferase or eGFP gene was linked to the Tat peptide via electrostatic interaction between the negatively charged plasmid and positively charged Tat [106,107]. Electrostatic charge interaction was also used to generate a complex between cationic CPPs and anionic siRNAs [108,109,110]. Readers are referred to an excellent review that summarizes the use of CPPs for nucleic acid delivery [111]. Recently, CPPs have been used as a mean for cell-specific delivery of viral genes [112] and to increase transduction efficiency. Both noncovalent binding (electrostatic interactions) and covalent binding (genetic engineering and chemical conjugation) have been employed to produce CPP-viral particles [113]. Traditional CPPs such as Tat, penetratin, and poly-arginine were the main CPPs used in the discussed studies. 

In a separate study, a bacteriophage displaying a CPP was used as a shuttle to deliver an adeno-associated virus (AAV) gene. AAV, cloned within the single-stranded genome of the bacteriophage [114,115,116,117], contained a mammalian transgene cassette encoding the cytomegalovirus (CMV) promoter-driven transgene, such as GFP and luciferase, as well as a polyA region that was flanked by inverted terminal repeats (ITRs) from AAV2 genome [116,117]. In the reported studies, Arg-Gly-Asp (RGD-4C) peptide targeting αv integrin receptors on vascular endothelial cells was displayed at N-terminus of phage PIII protein to facilitate AAV-phage cell entry. The incorporation of the ITRs of the AAV into phage genome has been shown to allow superior transgene expression in mammalian cell [118,119]. Depending on the inserted transgene, the phage genome size can be expanded to 13 Kb or higher, limiting phage amplification and assembly. 

### 2.7. Utilization of Oocytes to Access CPP Activity

Natural membranes of specialized cell types can offer valuable information about interaction of CPP and CPP-cargo with the cell membrane. The oocytes of the African frog *Xenopus laevis* were reported to express low level of ion channels and receptors [120]. The large size of *Xenopus* oocytes (diameter of up to 1.3 mm) allows easy dissection and electrophysiological manipulations [120]. In one study, oocytes were patch clamped under a two-electrode voltage-clamp to monitor changes in transmembrane current upon pore formation by amphipathic CPPs. Application of three amphipathic CPPs, including MPG, Pβ, and Pep-1, to the voltage-clamped oocytes resulted in increased transmembrane current that resembled channel-formation. Hence, the authors suggested that transmembrane pore formation was likely the mechanism for intracellular entry by the three peptides [47,48].

The large size of oocytes also allows microdissection and injection of various substances. Oocytes can synthesize an exogenous protein when a foreign messenger RNA is injected into their cytoplasm. Examples include neurotransmitter- and voltage-activated ion channels and G-protein coupled receptors [120,121,122]. Therefore, oocytes could be used as a model for membrane protein expression. Their simplified membrane system also allows the study of peptide-membrane interaction. The simplified version of oocytes includes lipid vesicles, which is the focus of next section. 

## 3. Lipid Vesicle-Based In Vitro Assays

The asymmetric lipid bilayers are patched with cholesterol and transmembrane proteins to provide the dynamic structure and elasticity of the membrane [123,124]. Since the early 1960s, artificial lipid membranes were used to study membrane lipid structure [125]. Today, they are broadly used to study peptide-membrane interaction and lipid reorganization under the conditions lacking endocytosis. General methods were developed to prepare different types of free-standing lipid vesicles [126]. These vesicles can vary in size and lamellar structure and can be synthesized as unilamellar, oligolamellar, and multilamellar vesicles. Lipid classes and their molecule-membrane interactions has been summarized in a 2017 publication by Rosilio. It is worth mentioning that, in most reported studies, mixed lipid models represent a better simulation of the complex biological membranes of cells in normal or diseased state [127]. The plasma membrane of eukaryotic cells are most frequently modeled by mixed bilayers of neutral and charged lipids at different molar fractions. Examples of zwitterionic and anionic lipids are dimyristoylphosphatidylcholine (DMPC) or palmitoyloleoylphosphatidylcholine (POPC) and dimyristoylphosphatidylglycerol (DMPG) or palmitoyloleoylphosphatidylglycerol (POPG), respectively. The molar fraction ratios between zwitterionic and anionic lipids has been varied (1:1, 9.5:0.5, 9:1, 8:2, 7.5:2.5, 3:1 mol/mol) depending on the experimental design. Lipids organize and coexist in solid and fluid phases [128]. DMPC and DMPG are considered as gel phase or ordered lipid, whereas POPC and POPG are referred to fluid phase or dis-ordered lipids [127]. Choice of ordered or disordered lipids may reflect the difference in phospholipid bilayer thickness. For example thickness of DMPC bilayer (carbonyl-to-carbonyl) is about 23 Å, whereas the POPC bilayer is 27 Å thick [129]. 

A considerable number of studies have been conducted with lipid vesicles to gain an insight in the mechanism of membrane binding and penetration of CPPs into intracellular space. It should be noted that key aspects of these studies (lipid components/architecture, CPP to lipid vesicle ratio, CPP concentration, lipid concentration, buffer composition, temperature, and pH) should be matched to physiological conditions to closely reflect biological systems. In addition to lipid composition, peptide to lipid (P/L) ratio can have a significant impact on the outcome of a CPP study. In fact, concentration-enhanced translocation has been reported for multiple CPPs in both cells and synthetic vesicles [130]. The observed increased translocation rates due to increased P/L ratio might suggest a translocation mechanism that is facilitated by peptide multimers and/or membrane perturbation, or both. Fuselier and colleagues have reported that translocation of LRLLRWC peptide was significantly reduced when P/L was varied from 1:100 to 1:500, whereas TP2 (PLIYLRLLRGQWC) showed an opposite behavior. This suggested that TP2 translocation might be driven by monomers and could be inhibited by lateral interactions in the membrane [49]. Utilization of lipid bilayers to study CPPs has advantages and limitations. Parameters that define lipid vesicle formation can be well controlled during experimental design, yet cell type specificity is difficult to achieve. CPPs and CPP-cargos might cross the lipid membrane without bilayer disruption. However, the cargo size can be restrictive in the absence of endocytosis.

### 3.1. Structural Change in CPPs upon Peptide/Lipid Interaction 

Many tools and techniques have emerged to study peptide/membrane interactions, including differential scanning calorimetry (DSC), plasmon waveguide resonance (PWR), NMR (solution and solid) [33,131,132], X-ray scattering [132,133], isothermal titration calorimetry (ITC), circular dichroism (CD), dual polarization interferometry (DPI) and mass spectrometry (MS) [18]. NMR and X-ray crystallography are capable of providing the residue-specific information. DPI and CD are simpler methods and can be used to measure absorption of optically active chiral molecules. Interaction of penetratin and R8K-biotin (RRMKWKKK(Biotin)-NH2) with a lipid bilayer was monitored by DPI to determine changes in mass per unit area and birefringence (an optical parameter representing bilayer order) with high sensitivity [30]. Both peptides bound strongly to anionic DMPC/DMPG and POPC/POPG, but not to neutral DMPC and POPC bilayers, indicating that electrostatic interaction between a lipid bilayer and CPP was required. CD has been used to monitor structural alterations in CPPs induced by the changes in environmental conditions (e.g., pH, temperature). CD spectra reflect the quantity of peptide secondary structure (α-helices, β-sheets, β-turns) [134], hence can be used to reveal functional plasticity of CPPs. For example, penetratin assumes a random coil configuration in solution. However, its configuration was altered in the presence of negatively charged vesicles. Interestingly, the extent of this alteration was dependent on the molar fraction of the charged lipids in the vesicles. The spectra were α-helical in the presence of low fractions of anionic lipids or low peptide/lipid ratios. On the other hand, it was suggested that penetratin assumes a β-sheet configuration in the presence of high fractions of anionic lipids or high peptide/lipid ratios. Comparable α-β transition was also observed if the composition of lipid vesicles were held constant and the concentration of penetratin was increased [31,32,33,34]. CD spectroscopy was also used to examine how different fluorophores influence the interaction of fluorophore-penetratin conjugates with membrane. The N-terminus of penetratin was conjugated to six different fluorophores (CF, TAMRA, RhB, NBD, MCA, and PBA) [135] and conformation of fluorophore-penetratin as well as nature of their interaction with membrane (POPC/POPG, 80:20) was assessed by CD. Interestingly, fluorophore–penetratin conjugates assume different conformations in solution compared to the unconjugated penetratin. Conjugation also increased α-helicity of penetratin upon membrane interaction. The two hydrophobic conjugates, RhB- and PBA-penetratin, caused a high membrane disturbance, indicated by calcein release from phospholipid vesicles. It was suggested that the observed increased membrane permeabilization and lipid removal in this study are mainly caused by fluorophore, raising a strong concern for fluorophore conjugation to CPPs. The assays investigating the secondary structure change of CPPs upon interaction with a lipid bilayer can be a valuable complementary approach to decipher mechanism of interaction of CPPs with the cell membrane.

### 3.2. CPP Translocation in Dye-Leakage Assay

Fluorescence techniques in the context of synthetic phospholipid bilayer have been used to analyze peptide interaction, translocation, and membrane perturbation. For this purpose, two strategies are commonly employed: outside-in and inside-out. In the outside-in approach, fluorescence signal induced by the dye labeled CPP is measured inside the vesicles. In the inside-out approach, dye is filled inside the vesicle and its leakage is measured upon CPP entry. Drin and co-workers labeled N-terminus of penetratin with a fluorescent NBD and used CD to monitor its conformation upon interaction with a mixer of POPC/POPG (95:5) small unilamelllar vesicles (SUVs). Although peptide interaction with the lipid bilayer was confirmed, spontaneous translocation of peptides was not observed, suggesting that NBD-penetratin might interact with cell surface components other than the lipids [27]. In a separate study, it was shown that penetratin could slowly translocate into a large unilamellar vesicle (LUV) instead of SUVs, indicating that the translocation of penetratin is depended on the phospholipid composition and requires a membrane potential [35]. A similar finding has shown that the fluorescence labeled octa-arginine derivatives could penetrate into HEK293 cells, but strictly accumulate in the vesicle membrane (POPC/POPG, 90:10) even at µM concentration. These studies have led the authors to suggest that the negative membrane potential is necessary for the CPPs to translocate inside the cell [43]. 

Enzymes were inserted in the lipid vesicles to improve assay specificity. Chymotrypsin (3uM) and terbium (Tb3+) entrapped in LUVs (POPC/POPG, 90:10) were used to investigate entry of CPP linked to an aminomethylcoumarin (AMC) conjugated tripeptide GQF (CPP-GQF-AMC). Upon entering, chymotrypsin cleavage site between Phe-AMC should be cut to release the AMC group, enabling its detection (excitation at 340 nm and emission at 440 nm for cleaved AMC) [136]. Addition of dipicolinic acid (DPA) to the vesicle’s external environment would result in Tb3+/DPA complex formation if Tb3+ was leaked due to membrane disruption. Peptides containing an LRLLR motif were screened for spontaneous membrane translocation in the context of synthetic lipid bilayer composed of POPC-LUVs-chymotrypsin [49,136]. In addition, a fluorophore-based assay was conducted to measure vesicle permeabilization. POPC-LUVs was prepared with entrapped ANTS, a fluorophore, and its quencher DPX, referred to as ANTS/DPX-POPC-LUVs. The vesicles were incubated with CPPs and any membrane leakage could be detected by ANTS fluorescence using a plate reader [49].

Translocation of CPPs that relies on high peptide concentrations possibly involves membrane disruption [137]. The colorimetric assays developed to exhibit color changes upon interactions of peptides and lipid membrane help the visualization of the membrane disruption. Phospholipids and the chromatic lipid-mimetic polymerized polydiacetylene (PDA) were prepared as vesicles. Following the addition of a CPP, the polymer could undergo concentration dependent blue-red transition if any structural perturbation was induced, suggesting peptide-membrane interaction or membrane disruption. Different degrees of color transition constitute an indication for peptides’ distinctive mode of interaction with the lipid vesicle interface [138,139].

### 3.3. CPPs Translocation and Membrane Potentials

Eukaryotic cells maintain a net negative potential inside the plasma membrane. Membrane potentials result from the transmembrane lipid asymmetry and can in turn govern the lateral segregation of lipids in the membrane. It was suggested that the modification of membrane potentials could affect CPP translocation [140,141]. 

In an attempt to create a negative charge inside the lumen, POPC-LUV vesicles were filled with 128 mM KCl and 128 mM NaCl was added to the external environment. A potassium-specific ionophore was embedded in the vesicles to create a negative potential inside the membrane. It was shown that an electrochemical potential could affect translocation of TP2 and its variants crossing the vesicle [49]. The dimension of GUVs resemble a cell and allows study of interaction of CPP with lipids including binding, penetration through the vesicle, and translocation to the GUV lumen [45,142,143]. Lin and colleagues reported that the membrane potential served as a driving force for the permeation of cationic CPP, such as 9R and Tat. They applied fluorescently labeled CPPs to GUVs with POPC/POPS (80:20) to monitor membrane crossing by fluorescence correlation spectroscopy (FCS). In addition, the researcher created a tunable membrane potential via the addition of a lipophilic ruthenium (II) complexed with Ru(C17)_2_^2+^ to induce hyperpolarization in the GUV membrane. The membrane hyperpolarization was driven by a chemical reaction and allowed photo-oxidation of potassium ferricyanide, Ru(C17)_2_^2+^. Ru(C17)_2_^2+^ by itself was shown not to alter the membrane permeability. Addition of Ru(C17)_2_^2+^ to the GUVs enhanced 9R permeation from 55% to 78%, possibly due to higher affinity of arginine to the phosphoserine head groups. Lipid vesicles do not bear negative membrane potentials. Therefore, the authors suggested that the membrane potential could be the major cause of discrepancies when CPP permeation is observed in the cellular versus synthetic vesicular [42]. 

### 3.4. CPPs Translocation in Plasma Membrane-Derived Vesicles

Giant plasma membrane vesicles (GPMVs), also called membrane blebs, are derived from the parental cells’ plasma membrane and are used as the model system for native plasma membrane [144]. The lumen of GPMVs contains cytoplasm but is free of cellular organelles and cytoskeleton, therefore it lacks energy fuels [145]. Readers are referred to a deep dive in GPMVs written by the Leventals [146]. The lipids of GPMVs were composed of phospholipid/cholesterol at a ratio of approximately 2:1 and retained the sophisticated complexity of the plasma membrane [147]. Saalik and colleagues generated GPMVs from RBL-2H3 and HeLa cells to study six known fluorescent labeled CPPs including Tat, penetratin, 9R, MAP, transportan, and TP10 in the absence of endocytotic processes [36]. Dye-CPPs were incubated with GMPVs and their penetration was quantified by flow cytometry and confocal microscopy. The authors showed that all tested CPPs accumulated into the lumen of GPMVs at 25 °C and lower temperatures. It should be noted that the preservation of membrane surface properties is the key step in GPMVs preparation [146]. Liu and co-workers pointed out that the chemical vesiculants (e.g., formaldehyde and dithiothreitol) and osmotic buffers that are required for vesicle formation could disrupt membrane structure. They herein developed a high yield nanomaterial-assisted strategy that used light irradiation to generate GPMVs in biocompatible medium such as Dulbecco's Modified Eagle's Medium [148]. Further optimization of GPMVs has expanded their application as an intact membrane with lipid and protein complexity of mammalian plasma membrane. Artificial plasma membranes are also used to study protein function. For example, sophisticated design of ion channels were incorporated in lipid vesicles to develop sensing applications [149]. Moreover, computational methods such as simulation of interaction of CPPs with artificial lipid bilayers have been utilized. Molecular dynamics simulations can provide means to gain insight into the peptide/membrane interactions and various energy-independent mechanisms. A more extensive review of the molecular dynamics application for CPPs can be found in review written by Reid [150]. 

## 4. Concluding Remarks

Tremendous effort has been made for the discovery and characterization of novel CPPs for efficient and specific cytoplasmic delivery. Fundamental understanding of the mechanism of cell entry, endosomal escape, and interaction of CPPs with the cell membrane are critical for their advancement in the clinic. Biological relevance of the cell lines used in the in vitro assays should be taken into consideration to preserve in vitro–in vivo connectivity. In this review, we have summarized current strategies that were utilized or could be employed in evaluating CPP-cargos. Since each technique bears its limitations, exploiting orthogonal strategies is recommended to identify the most efficient CPPs as vehicles for therapeutic cargo delivery. 

## Figures and Tables

**Table 1:** List of CPPs used in various assays.

CPP	Sequence	Length	MW	PI	Charge at pH 7	Ref.
Penetratin	RQIKIWFQNRRMKWKK	16	2246.76	12.8	6.94	[27,28,29,30,31,32,33,34,35,36]
Tat _(48–60)_	GRKKRRQRRRPQ	12	1621.92	13.18	7.94	[23,28,29,36,37,38,39,40,41,42]
Tat _(49–57)_	RKKRRQRRR	9	1339.62	13.18	7.94	
Transportan	GWTLNSAGYLLGKINLKALAALAKKIL	27	2841.48	10.7	3.94	[28,36]
MAP (KLAL)	KLALKLALKALKAALKLA	18	1877.47	11.14	4.94	[28,36]
R8K	RRMKWKKK	8	1160.5	12.52	5.94	[30]
8K	KKKKKKKK	8	1043.41	11.39	7.94	[38]
8R	RRRRRRRR	8	1267.52	13.33	7.94	[43]
9R	RRRRRRRRR	9	1423.7	13.38	8.94	[29,36,42,44,45]
Ypep	YTFGLKTSFNVQ	12	1404.59	9.19	0.94	[46]
MPG	GALFLGFLGAAGSTMGAWSQPKKKRKV	27	2807.36	11.85	4.94	[47,48]
Pep-1	KETWWETWWTEWSQPKKKRKV	21	2848.26	10.36	2.94	[48]
Pβ	GALFLGFLGAAGSTMGAWSQPKKKRKV	27	2807.36	11.85	4.94	[48]
TP2	PLIYLRLLRGQWC	13	1630.01	9.48	1.88	[49]
TP10	AGYLLGKINLKALAALAKKIL	21	2182.76	10.7	3.94	[36,45]

## Abbreviations

CPP	cell-penetrating peptide
GFP	green fluorescence protein
eGFP	enhanced green fluorescence protein
YFP	yellow fluorescence protein
eYFP	enhanced yellow fluorescence protein
RFP	red fluorescence protein
DMPC	dimyristoylphosphatidylcholine
DMPG	dimyristoylphosphatidylglycerol
DOPC	dioleoylphosphatidylcholine
POPC	palmitoyloleoylphosphatidylcholine
POPG	palmitoyloleoylphosphatidylglycerol
POPS	palmitoyloleoylphosphatidylserine
SUV	small unilamelllar vesicle
LUV	large unilamellar vesicle
GUV	giant unilamellar vesicle
GPMV	giant plasma membrane vesicle
MLV	multilamellar vesicle
CD	circular dichroism
DPI	dual polarization interferometry
NBD	N-(7-nitro-2,1,3-benzoxadiazol-4-yl)glycine
TAMRA	5(6)-carboxytetramethylrhodamine
CF	5(6)-carboxyfluorescein
RhB	N-(9-(2-carboxyphenyl)-6-(diethylamino)-3Hxanthen-3-ylidene)-N-ethylethanaminium
MCA	(7-methoxycoumarin-4-yl)acetic
PBA	1-pyrenebutyric acid
